# Efficiency of a multidisciplinary team care approach through a short hospitalization of patients with poorly controlled diabetes mellitus: a 12 months prospective monocentric study

**DOI:** 10.11604/pamj.2022.41.192.23965

**Published:** 2022-03-09

**Authors:** Ach Taïeb, Lemdjo Gaëlle, Ducloux Roxane, Wojewoda Perrine, Albentosa Marion, Bougeniere Fleur, Leriche Zoé, Leveque Aurélie, Dennetiere Solen, Dutrieux Patricia, Averous Véronique

**Affiliations:** 1 Department of Endocrinology and Diabetology, Douai Hospital Center, Douai, France,; 2 University of Sousse, Faculty of Medicine of Sousse, 4000, Sousse, Tunisia,; 3 University Hospital Farhat Hached, Sousse, Tunisia,; 4 Faculty of Medicine and Biomedical Sciences, University of Yaoundé I, Yaoundé, Cameroon

**Keywords:** Diabetes, professional education, nursing role, nutritional care, primary health care, health care education

## Abstract

A multidisciplinary team is composed of various healthcare professionals that ensure a multifaceted approach on a group of patients. Standards for diabetes medical care note the importance of multidisciplinary diabetes care teams. We applied our model of multidisciplinary approach by structuring it in a determined five days hospitalization. The aim of this study was to determine if the interdisciplinary approach applied on a short hospitalization is of benefit in patients with poorly controlled diabetes mellitus. Sixty-seven patients were included and ensured a short hospitalization in which they received a multiple educational advice and a treatment adaptation. Sixty-one patients out of 67 (91%) were retained for evaluation with sufficient data at one year, i.e. 9% of patients with poor compliance. Evolution in glycosylated haemoglobin (HbA1c), weight and treatments was analyzed. After a 12 months follow-up, we observed significant improvement in HbA1c (-1.73%; p < 10^3^) without weight loss (BMI=-0.42 kg/m^2^), p=0.28). HbA1c mean levels correlated negatively to body mass index (BMI) during the regular follow-up (r=-0.22, p=0.05). More than 90% of patients with poorly controlled diabetes mellitus responded to the multi-disciplinary approach with a decrease in HbA1c.

## Introduction

The number of patients diagnosed with diabetes is increasing across the globe. In future projections, the diagnosed diabetes incidence will increase from about 8 cases per 1,000 in 2008 to about 15 in 2050 [1]. According to the announcement by the American Diabetes Association (ADA) and European Association for the Study of Diabetes (EASD) consensus [2], providing patient-centered care with a multi-disciplinary team is essential to effective diabetes management. In the common comprehension of the multi-disciplinary team approach, it includes a variety of healthcare professionals [3,4]. In our department, a therapeutic program has been built to fit in a five days´ hospitalization, according to organizational constraints. Some health providers may doubt of the efficiency of hospitalizing patients to help equilibrate their diabetes. In the following study, we analyzed the impact of a multi-disciplinary teamwork in managing diabetes, through a short duration hospitalization, by assessing its efficiency after 12 months´ follow-up.

## Methods

**Study design and setting:** we conducted a prospective observational study in the Endocrinology and Diabetology Department of the Hospital Center of Douai. The study lasted twelve months since the programmed hospitalization date of each patient. This time frame was considered adequate as to allow patients´ visits to the hospital or their own general practitioner, and assess medium term efficacy of the educational program instead of short-term motivation only.

**Study population:** all patients with poor control of diabetes, defined as an off-target HbA1c for patients´ diabetes age and comorbidities [5], were included in the study. The only patients considered at the end of the study were those who got all the data at the end of their complete year follow-up: HbA1c and weight for each 3 months till 1 year. Patients were followed even by their general practitioner or by the diabetologists of Douai´s Hospital Center. All types of diabetes were included. Patients were separated in various sized groups, ranging from 6 to 10, hospitalized from Monday to Friday of the same week. The starting follow-up of each patient began from the end of the hospitalization, till twelve months from the day of exit. Patients suffering from a severe state of illness that may interfere with the good follow-up or the glycemic balance such as final stage cancers, cognitive diseases, and severe physical incapacities were excluded. The main aim was to ensure that all the patients would be able to apply the educational skills provided by the doctors, dieticians and medical sport coaches.

**Study protocol:** special emphasis was put in patient education, aimed to improving compliance and changing lifestyle. A structured program was adapted to particular life habits, physical possibilities and comorbidities. The patients were scheduled for a first visit with the diabetologist, the dietician and the diabetes nurse educator. At this point, the patient´s educational and therapeutic goals were established. Counseling by the dietitian was performed individually and in accordance with the Clinical Practice Recommendations of the ADA. The diabetes nurse educator taught patients the principles of diabetes and introduced them to the activities of performing and interpreting the results of self-glucose monitoring. Talk groups were proposed to share life experiences with diabetes. Participants were asked open-ended questions about macrovascular and microvascular diabetes complications and any adverse events (for example, hypoglycemia), serious adverse events or hospitalizations. Two medical sport coaches taught the main principles of a physical activity adjusted to the medical history of the patients. The evaluation of the capability in sport´s exercise was evaluated using self-report questionnaires to measure qualitatively the physical activity [6]. All able patients were offered to join a physical activity session during their stay. Group sessions were even proposed beyond hospitalization. Our team was covered by two medical psychologists specialized in the follow-up of chronic behavioral diseases, eating disorders, and helped patients in stressful psychological situations. The psychologists also asked participants to rate their HRQL (using the EuroQol-5D visual analogue scale, with scores ranging from 0 to 100) [7] and with the Hospital Anxiety and Depression scale [8] to assess the symptom severity of anxiety disorders and depression in diabetic patients.

A treatment protocol was applied. A patient´s treatment was set to reach target and was adjusted according to response. Intensified glycemic checking allowed physicians to better adapt and individualize the treatment, according to glycemic diurnal and nocturne profile. For the whole week of hospitalization, medical actors relayed themselves to interact, discuss, inform and educate the patients. Meanwhile, the anti-diabetic treatment was adapted to reach a best glycemic target. After the exit from the hospital, all patients were scheduled for regular follow-up visits by our own doctors or their private practitioner and for laboratory analysis at 3, 6, 9 and 12 months. Clinical and laboratory data and drug prescriptions at the beginning and the end of 12 months were retrieved from the patients´ records and analyzed.

**Endpoints:** the study had two main endpoints: A) modifications of HbA1c at the end of the study. We analyzed the whole cohort´s HbA1c rates as mean ± standard deviation, and observed its variability. B) Subjects´ BMI at the end of 12 months of follow-up. We analyzed the whole cohort´s BMI variability as mean ± standard deviation.

**Secondary endpoints:** A) percentages of patients that met successful endpoints, meant by a lowering in HbA1c and in weight. B) The percentage of patients who were considered to be compliant to the study protocol if they returned for the follow-up visit at 12 months and the number of patients not included because of a lack of data.

**Statistical analysis:** it was performed using the IBM SPSS Version 23.0 (IBM Inc) program. Continuous variables were described by their means and standard deviations. Chi-square tests were used for qualitative variables. Correlation analysis was conducted with Pearson´s analysis for parametric variables. We used a Student paired samples T Test to compare means. A p value <0.05 was considered as statistically significant.

**Ethical considerations:** a written informed consent was obtained from all the patients before the beginning of the study.

**Informed consent:** a written informed consent was obtained from all the patients before the beginning of the study.

## Results

**Figure 1:**
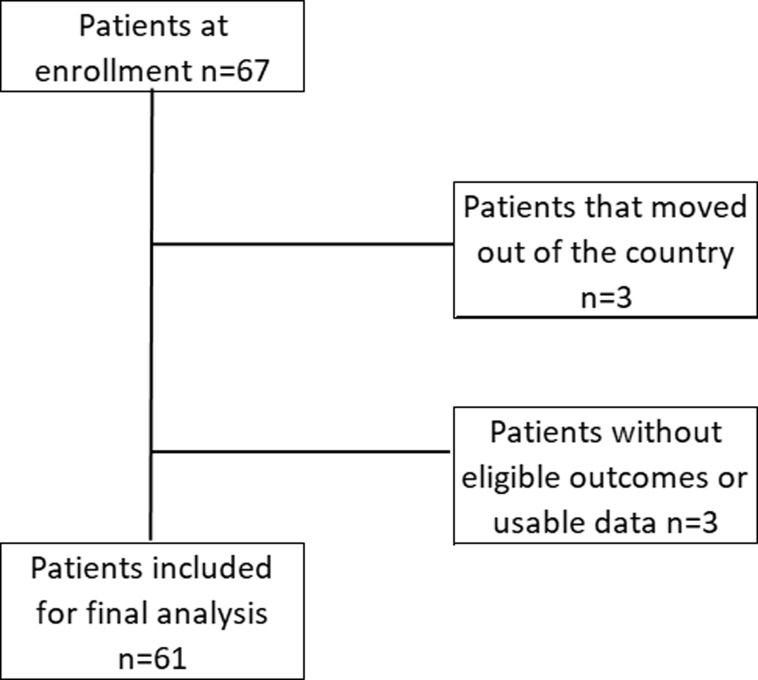
study profile and final included patients

**General characteristics:** of 67 participants who were screened in the beginning of the study, 61 patients (91%) were eligible by providing all the necessary data and because they attended all their meetings with our doctors or their own physicians (Figure 1). Sixty-one patients (32 men, 29 women) were included in the study after oral and informed consent. Types of diabetes represented were: 51 patients with type 2 diabetes (83.6%), 8 patients with type 1 diabetes (13.1%) and 2 patients with pancreatic diabetes (3.3%). The mean age of participants was 60.4±16.2 years old. At the beginning of the study, all the patients were receiving anti-diabetic medications. At enrollment, baseline HbA1c level was 10.05±1.4% and mean BMI was 31.5±7.5 kg/m^2^). The Table 1 shows the repartition of BMI and patients´ characteristics. Concerning anti-diabetic treatment, 34.4% (n=21) of the patients were under non-insulinic medications (oral treatments and/or GLP1 analogs) and 65.5% (n=40) were under insulin associated or not with oral medications (treatments are detailed in Table 2). At the end of the hospitalization, 62.3% (n=39) of the patients had their treatment changed (p<0.001) (Table 2).

**Table 1:** patients´ baseline characteristics at inclusion

Variables	Parameters	Frequency % (n)
**Sex**	men	52.5% (n=32)
	Women	47.5 (n=29)
**Bm^2^I**		
	< 24.9 kg/m^2^	16.4% (n=10)
	25-29.9 kg/m^2^	32.8% (n=20)
	30- 34.9 kg/m^2^	23% (n=14)
	35-39.9 kg/m^2^	14.8% (n=9)
	> 40 kg/m^2^	13.1% (n=8)
**HbA1c**		
	<8%	22.9% (n=14)
	8 - 9.9%	31.1% (n=19)
	10 - 11.9%	32.7% (n=20)
	12-13.9%	11.5% (n=7)
	>14%	1.6% (n=1)

**Table 2:** anti-diabetic protocols before and after hospitalization

Variables	Parameters	Before Hospitalization % (n)	After treatment change % (n)
**Schema Protocols**	OAD only	29.5% (n=18)	8.2% (n=5)
	OAD + GLP-1	4.9% (n=3)	11.5% (n=7)
	Insulin only	44.3% (n=27)	49.2% (n=30)
	OAD + Insulin	18% (n=11)	24.6% (n=15)
	Insulin + GLP-1	1.6% (n=1)	1.6% (n=1)
	OAD + Insulin + GLP1	1.6% (n=1)	4.9% (n=3)
Types of oral treatments(*)			
	Metformin	34.4% (n=21)	42.6% (n=26)
	Sulfonylureas	26.3% (n=16)	16.4% (n=10)
	Gliptin	18% (n=11)	9.8% (n=7)
	Glinide	9.8% (n=7)	3.3% (n=6)
**Insulin Protocols**			
	Basal	14.8% (n=9)	14.8% (n=9)
	Basal Plus	1.6% (n=1)	3.3% (n=2)
	Basal Bolus	34.4% (n=21)	48.8% (n=29)
	Premix Insulins	6.6% (n=4)	6.6% (n=4)
	Insulin pump	8.1% (n=5)	6.6% (n=4)

OAD: oral antidiabetic drugs; GLP1-A: Glucagon-like peptide-1 analogue; (*) : glitazones and gliflozins are not available in France.

**Figure 2:**
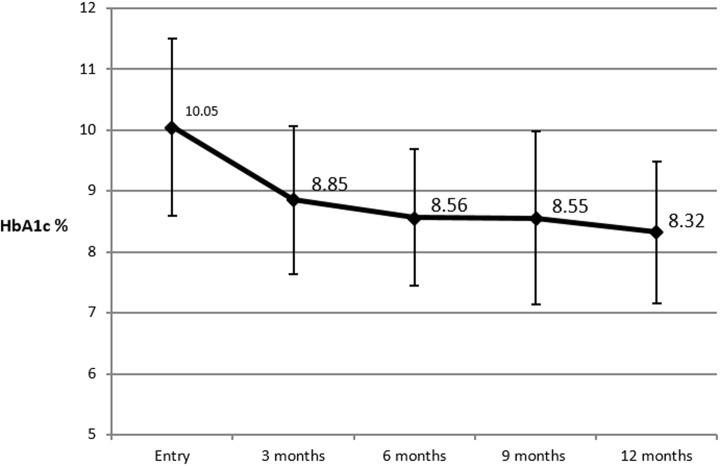
mean HbA1c levels and evolution through one year of regular follow-up

**HbA1c analysis:** at the end of the follow-up, HbA1c decreased significantly from an initial value of 10.05±1.4% to a one-year HbA1c value of 8.3±1.1% (mean HbA1c decrease=-1.7%, p < 0.001). The main decrease was seen in the three first months after hospitalization (p<0.001) and maintained thereafter (Figure 2). Amongst the whole cohort, 57 patients (93.4%) achieved a decrease in their HbA1c (p<0.001).

**Figure 3:**
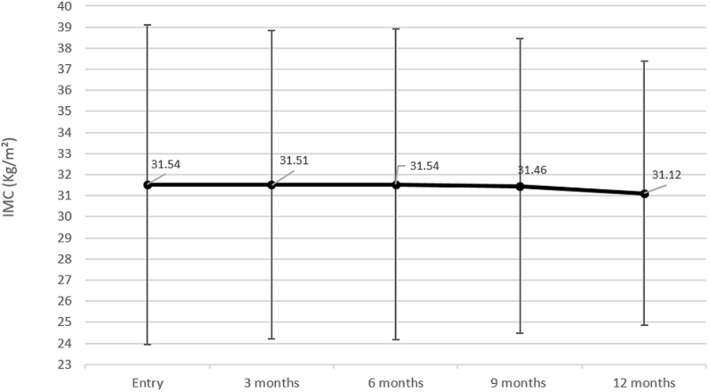
mean body mass index and evolution through one year of regular follow-up

**BMI analysis:** analyzing the evolution of BMI at the end of the follow-up, the decrease was not significant, with an initial value of 31.5±7.58 Kg/m^2^ to a final value of 31.1±6.2 Kg/m^2^ (mean BMI decrease=-0.42 kg/m^2^), p=0.28) (Figure 3). HbA1c mean levels correlated negatively to BMI during the regular follow-up (r=-0.22, p=0.05). The correlation indicates a tendency to lose weight for patients improving HbA1c. Amongst the cohort at the end of the follow-up, 57.5% achieved a decrease in their BMI. The combined success criterion which is the associated lowering of HbA1c and BMI was encountered in 57.8% of the whole Cohort.

## Discussion

In this study, we reported the effectiveness of a multi-disciplinary intervention in the treatment of poorly controlled diabetes mellitus. Our results showed a successful achievement of the main endpoint which was a decrease of the HbA1c through a regular follow-up since the short hospitalization. Our significant mean decrease was of -1.73% after twelve months´ follow-up, which seems to be similar to other results in the literature [9]. In addition to that, 93.4% of our patient´s cohort achieved a decrease in their HbA1c, which was significant and even superior to other studies´ results [9]. Our second main point, which was the decrease of the BMI, was not achieved. This could be explained by some hypotheses, among them, the fact that the follow-up duration was of one year only and that the decrease in BMI was in a continuing long process. Also, the variability of the treatment protocols may interfere with the weight loss, as we showed in our cohort that a majority of our patients was under insulin therapy, known to increase the weight if overused [10]. Analyzing our secondary endpoints, the percentage of patients who were considered to be compliant to the study protocol was of 91%. In other studies, the compliance was evaluated at 85% which is a similar result compared to our cohort [9].

The WHO declared that interprofessional education is valuable to accomplish the patient-centered care, asking for healthcare workers from different professions to learn from each other towards developing a more comprehensive care plan for diabetic patients [11]. A study by Li *et al* showed that participation in diabetes education and training within the first year after diagnosis is strongly recommended and shown positive influence on the future evolution of glycemic balance [12]. In our study, improving HbA1c for our patients arise from collaboration between physicians, nutritionists, diabetes nurse educators, psychologists and medical coaches. The role of physicians within the multidisciplinary collaborative care model was primarily in monitoring, counseling, assessing and optimizing medications of patients. Physicians used also to recommend lifestyle modifications, managing comorbidities concurrently with diabetes management and take part in future continuous medical education [13]. Nurses are crucial in providing good patient care and promoting self-care management. They work wholly in diabetes care and may be employed in a variety of care settings such as giving prevention advice, using behavior change and health coaching techniques, promoting self-care, identifying and treating hypoglycemia and hyperglycemia and the learning of injectable therapies´ use [14]. The combined partnership of nurse practitioners and physicians led to a mean reduction of 0.68% in HbA1c in patients [15]. Any approach to meal planning should be individualized considering the health status, personal preferences, and ability of the person with diabetes to sustain the recommendations in the plan [16,17]. Studies of nutritional interventions showed reductions in HbA1C of 0.3% to 2.0% in patients as well as improvements in medication doses and quality of life [17].

Physical exercise has been shown to improve blood glucose control, reduce cardiovascular risk factors, contribute to weight loss, and improve well-being [18]. Basic evidence supports that diabetics, should be encouraged to reduce the amount of time spent being sedentary by walking or performing other physical activities [19,20]. Medical psychologist is primordial in every multi-disciplinary team approach and has shown efficiency in addressing psychosocial issues in diabetic patients and emotional regulation [21,22]. A systematic review and meta-analysis showed that psychosocial interventions modestly but significantly improved HbA1C and mental health outcomes [23]. The strength of our study is that it showed the efficacy of a 5 days´ hospitalization in lowering HbA1c, through a structured educational program combined with treatment adaptations. It was a prospective study with a long term follow-up. However, the main limitation was the low number of participants in the total follow-up. The short hospitalization may be a solution to several departments lacking in the education process. Further studies are needed, comparing in patients with similar outpatients who did not beneficiate from the program.

## Conclusion

We showed that a short structured hospitalization of poorly controlled diabetic patients induces a 1.73% decrease in mean HbA1c, persistent one year after intervention, without weight gain. More than 93% of the subjects responded to the interdisciplinary educational and therapeutic approach with a decrease of HbA1c. The main originality of our work was the use of a short hospitalization´s protocol to permit a more organized multi-disciplinary team approach. Future studies must focus on how to decrease weight in overweight patients. Medico-economic analyzes and comparisons with outpatients would also be contributive.
